# Supplementary material for: Loss of Ku’s DNA end binding activity affects telomere length via destabilizing telomere-bound Est1 rather than altering TLC1 homeostasis
Source: Sci Rep. 2019 Jul 23;9:10607. doi: 10.1038/s41598-019-46840-2 (PMC6650470; doi:10.1038/s41598-019-46840-2)
Supplement: Supplementary file 1 — Supplementary Information and Figures S1–S18 [file 41598_2019_46840_MOESM1_ESM.pdf]

Supplementary Information

Loss of Ku's DNA end binding activity affects telomere length via destabilizing telomere-bound Est1 rather than altering TLC1 homeostasis

Laramie D. Lemon[1,2], Danna K. Morris[2], and Alison A. Bertuch[1,2,*]

[1]Graduate Program in Integrative Molecular and Biomedical Sciences, Baylor College of Medicine, Houston, TX, 77030, USA

[2]Department of Pediatrics, Baylor College of Medicine, Houston, TX, 77030, USA

[*]Corresponding Author

Alison A. Bertuch, MD, PhD

1102 Bates, FC 1200

Houston, TX, 77030

Tel: 832-824-4579

FAX: 832-825-4651

abertuch@bcm.edu

Supplementary Table S1. Strains used in the study

STRAIN	GENOTYPE	SOURCE
YAB289	*MAT**a** ura3-52 lys2-801 ade2-101 trp-Δ1 his3-Δ200 leu2-Δ1*	[1]
YAB200	YAB289 *yku70Δ::KAN^R*	This study
YAB470	YAB289 *cdc13Δ::NAT^R yku70Δ::HPH^R* pVL438	[1]
YAB471	YAB289 *cdc13Δ::NAT^R* pVL438	[1]
YAB540	YAB289 *yku70-R456E*	This study
YAB620	YAB289 *cdc13Δ::NAT^R yku70-R456E* pVL438	[1]
YAB621	YAB289 *yku80-135i*	[1]
YAB766	YAB289 *yku80Δ::NAT^R*	[1]
YAB852	YAB289 *tlc1Δ::KAN^R* pSD120	This study
YAB928	YAB289 *CDC13-G6-(FLAG)$_3$::KAN^R EST1-(MYC)$_{13}$::HIS3 yku80Δ::HPH^R*	This study
YAB930	YAB289 *CDC13-G6-(FLAG)$_3$::KAN^R EST1-(MYC)$_{13}$::HIS3*	This study
YAB936	YAB289 *EST1-(MYC)$_{13}$::HIS3*	This study
YAB937	YAB289 *CDC13-G6-(FLAG)$_3$::KAN^R EST1-(MYC)$_{13}$::HIS3 yku80-135i*	This study
YAB958	YAB289 *CDC13-G6-(FLAG)$_3$::KAN^R EST1-(MYC)$_{13}$::HIS3 yku70Δ::KAN^R*	This study
YAB959	YAB289 *EST1-(MYC)$_{13}$::HIS3 yku70-R456E*	This study
YAB961	YAB289 *CDC13-G6-(FLAG)$_3$::KAN^R EST1-(MYC)$_{13}$::HIS3 yku70-R456E*	This study
YAB1021	YAB289 *sir4Δ::KAN^R*	This study
YAB1023	YAB289 *tlc1Δ48*	This study
YAB1024	YAB289 *yku80-L111R*	This study
YAB1025	YAB289 *yku80-L115A*	This study
YAB1027	YAB289 *EST1-(MYC)$_{13}$::HIS3 YKU80-(FLAG)$_3$::KAN^R*	This study
YAB1028	YAB289 *EST1-(MYC)$_{13}$::HIS3 YKU80-(FLAG)$_3$::KAN^R yku70-R456E*	This study

Supplementary Table S2. Plasmids used in this study

**Plasmid**	**Genotype**	**Source**
pAB198	*CEN TRP1 yku70-R456E*	[2]
pAB830	*2μ URA3 ADH1-TLC1*	[1]
pAB889	*CEN URA3 yku80-135i*	This study
pRS414	*CEN TRP1*	[3]
pRS416	*CEN URA3*	[3]
pRS426	*2μ URA3*	[3]
pSD120	*CEN URA3 TLC1*	[4]
pVL438	*CEN URA3 CDC13*	[5]
pVL648	*CEN LEU2 CDC13*	[5]
pVL1057	*CEN TRP1 YKU70*	[6]
pVL1069	*CEN URA3 YKU80*	[7]
pVL1091	*CEN LEU2 CDC13-EST1*	[5]

References

1. Williams, J.M., et al., *The principal role of Ku in telomere length maintenance is promotion of Est1 association with telomeres.* Genetics, 2014. **197**(4):1123-36.
2. Lopez, C.R., et al., *Ku must load directly onto the chromosome end in order to mediate its telomeric functions.* PLoS Genet, 2011. **7**(8):e1002233.
3. Christianson, T.W., et al., *Multifunctional yeast high-copy-number shuttle vectors.* Gene 1992. **110**(1):119-22.
4. Diede, S. J., and D.E. Gottschling, *Telomerase-mediated telomere addition in vivo requires DNA primase and DNA polymerases alpha and delta.* Cell, 1999. **99**(7):723-33.
5. Evans, S.K. and V. Lundblad, Est1 and Cdc13 as comediators of telomerase access. Science,1999. **286**(5437):117-20.

6. Ribes-Zamora, A., et al., *Distinct faces of the Ku heterodimer mediate DNA repair and telomeric functions.* Nat Struct Mol Biol, 2007. **14**(4):301-7.
7. Bertuch, A.A. and V. Lundblad, *The Ku heterodimer performs separable activities at double-strand breaks and chromosome termini.* Mol Cell Biol, 2003. **23**(22):8202-15.

Supplementary Methods

Protein solubility experiments

Strains were grown at 28°C in 50 mL of YPD media to OD_{600}=0.8. Extracts were lysed in TMG (10 mM Tris-HCl, pH 8.0, 1 mM $MgCl_2$, 10% glycerol, 0.1 mM EDTA) plus 50 mM NaCl, PMSF (1:10 dilution), and Set III Protease inhibitor cocktail (Calbiochem, Millipore, 1:10 dilution) using acid washed glass beads. The supernatant (soluble fraction) was collected by centrifuging at 14000 RPM for 15 minutes at 4°C. The remaining cell debris pellet was resuspended in a solubilization buffer (20mM $NaPO_4$ buffer pH 8.0, 300 mM NaCl, 2% SDS, 2 mM DTT, 1% Triton X-100), PMSF (1:10 dilution), and Set III Protease inhibitor cocktail (Calbiochem, Millipore, 1:10 dilution) and the supernatant (insoluble fraction) was collected by centrifuging at 14000 RPM for 15 minutes at 4°C. Equal volume of soluble and insoluble fraction was run on a 7.5% polyacrylamide gel, transferred to an Immobilon-FL PVDF membrane (Millipore), and probed with α-FLAG (Sigma F7425, 1:1000 dilution), α-myc (Sigma M4439, 1:5000 dilution), and α-PGK (Abcam ab113687, 1:5000 dilution) primary antibodies. Percent Cdc13 and Est1 was calculated per fraction.

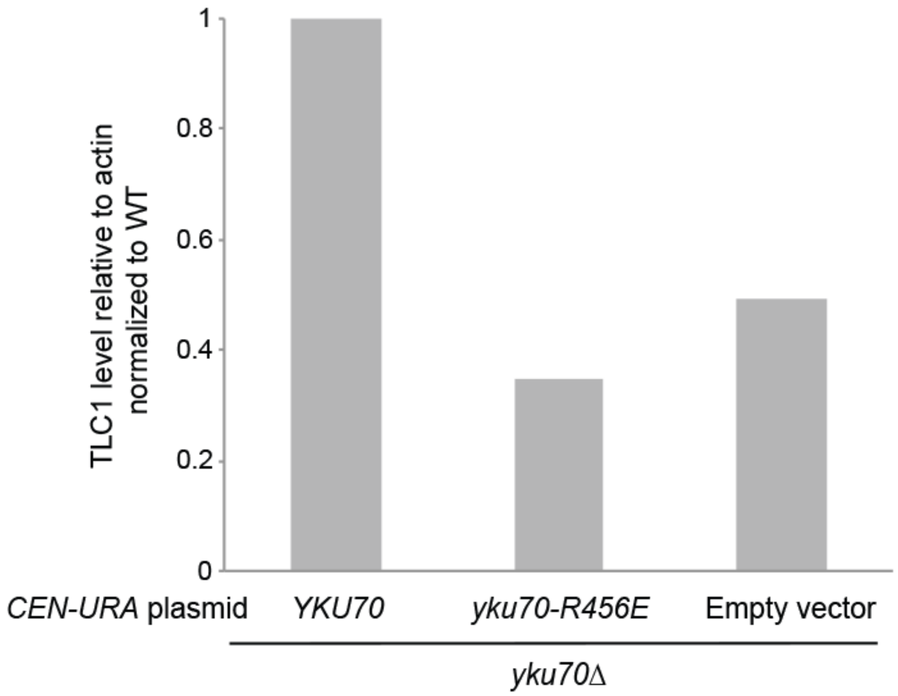

Supplementary Figure S1. ***YKU70*** **restores TLC1 levels in a** ***yku70*Δ strain while the** ***yku70-R456E*** **mutant fails to rescue TLC1 levels.** Quantification of TLC1 by RT-qPCR in asynchronous *yku70*Δ cells tranformed with indicated plasmids. TLC1 levels were quantified relative to actin RNA and normalized to levels obtained for the *YKU70 CEN-URA* transformed (WT) cells.

Figure-S2 (Bertuch)

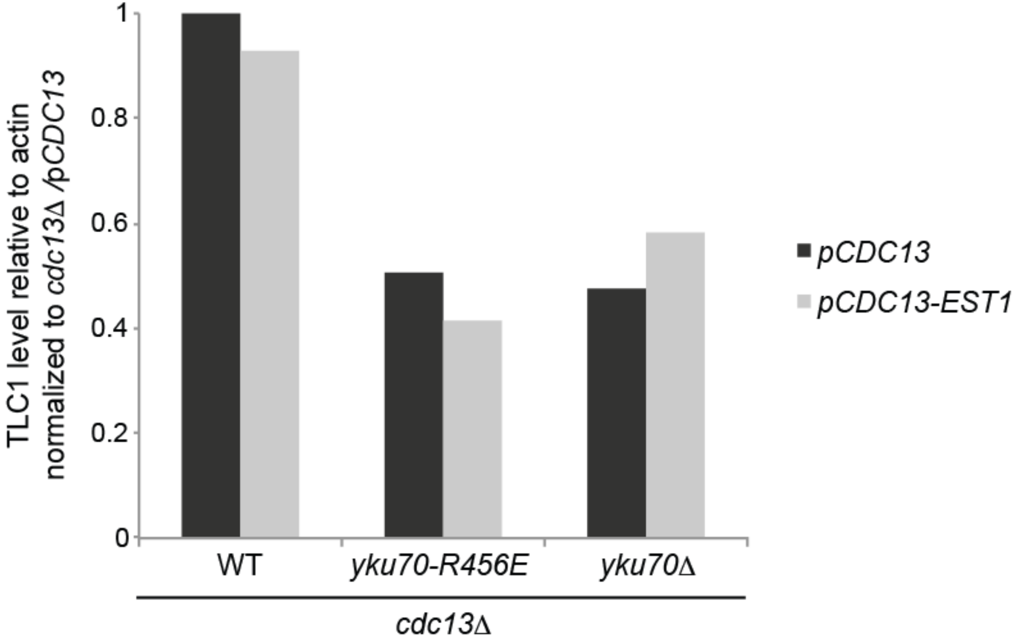

Supplementary Figure S2. TLC1 levels are not stabilized in Ku mutant strains expressing a *CDC13-EST1* fusion. RT-qPCR of TLC1 in asynchronous *cdc13Δ YKU70* (WT), *cdc13Δ yku70-R456E* and *cdc13Δ yku70Δ* strains expressing plasmid-borne *CDC13* or *CDC13-EST1* fusion.

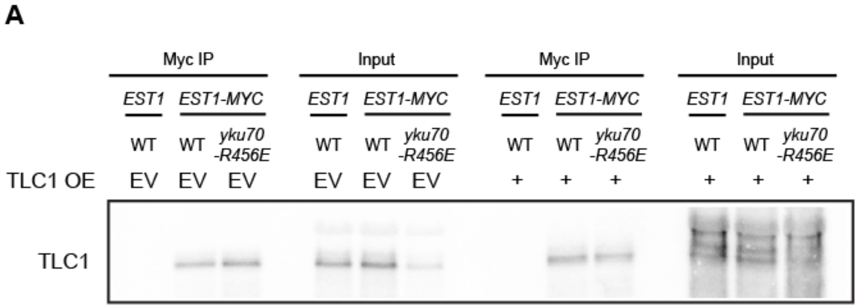

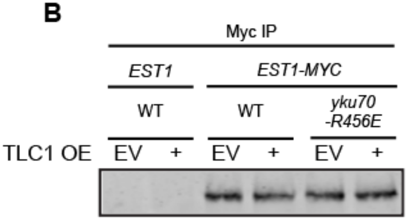

Supplementary Figure S3. Est1-TLC1 interaction is not reduced in a *yku70-R456E* strain, with or without TLC1 overexpression. Whole cell lystates from asychronous cultures of strains with EST1 or EST1-MYC, YKU70 (WT) or yku70-R456E, and carrying either EV or TLC1 OE 2 micron plasmids, were immunoprecipitated with anti-myc and examined by northern blot for co-IP of TLC1 (A) or western blot to detect Est1-myc (B). Full-length blots are presented in Supplementary Figure S15.

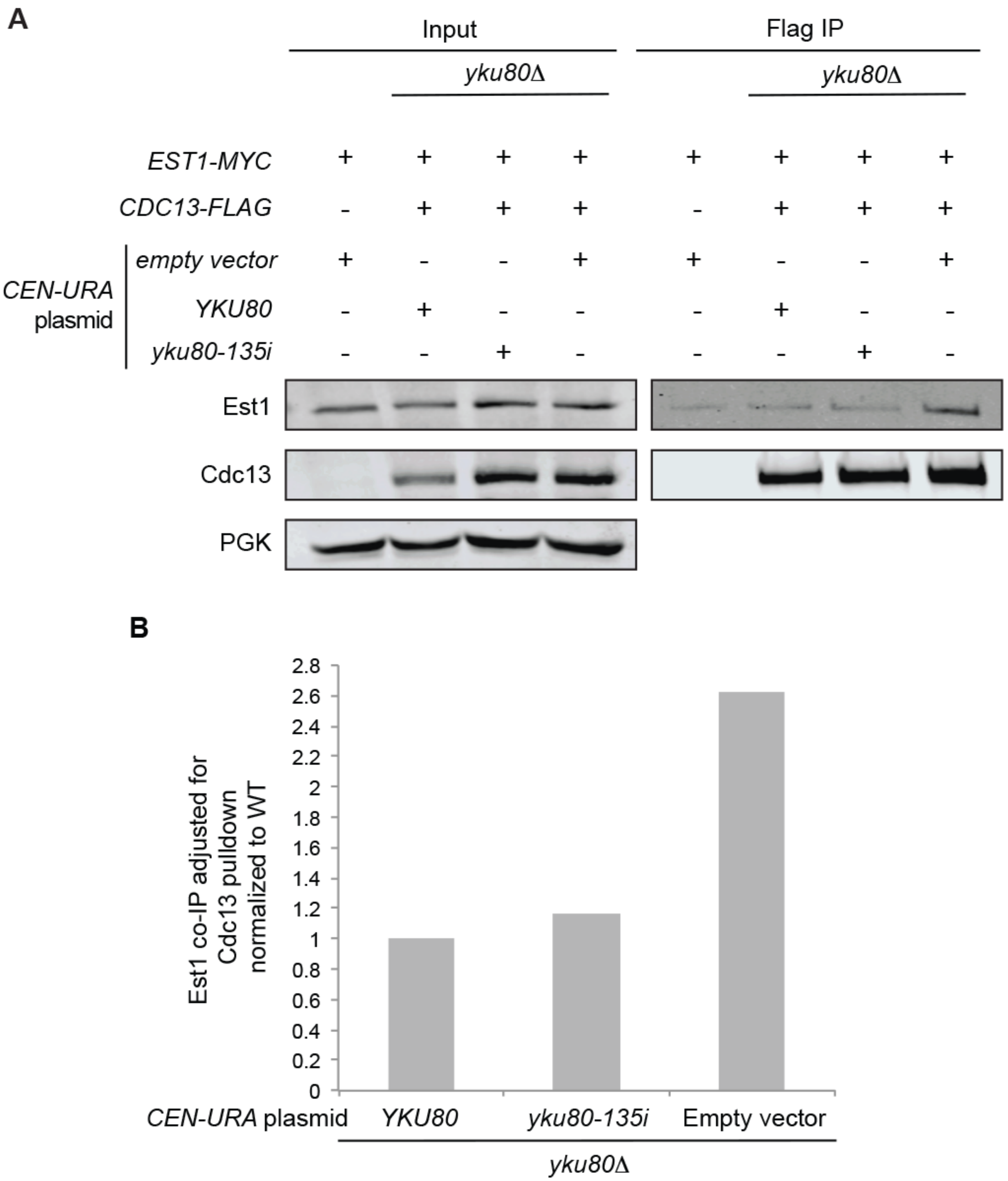

Supplementary Figure S4. The Est1:Cdc13 interaction is rescued with *YKU80* plasmids. (A) Co-immunoprecipitation of Est1-myc with Cdc13-FLAG in *yku80Δ* strains supplemented with indicated plasmids. Anti-FLAG immunoprecipitations were performed with whole cell lysate of asynchronous strains. Immunoprecipites and inputs were analyzed by western blotting with α-myc (Est1) and α-FLAG (Cdc13). Inputs were also probed with α-PGK for loading. Full length blots are presented in Supplementary Figure S14. (B) Quantification of Est1 associations relative to Cdc13 pull-down.

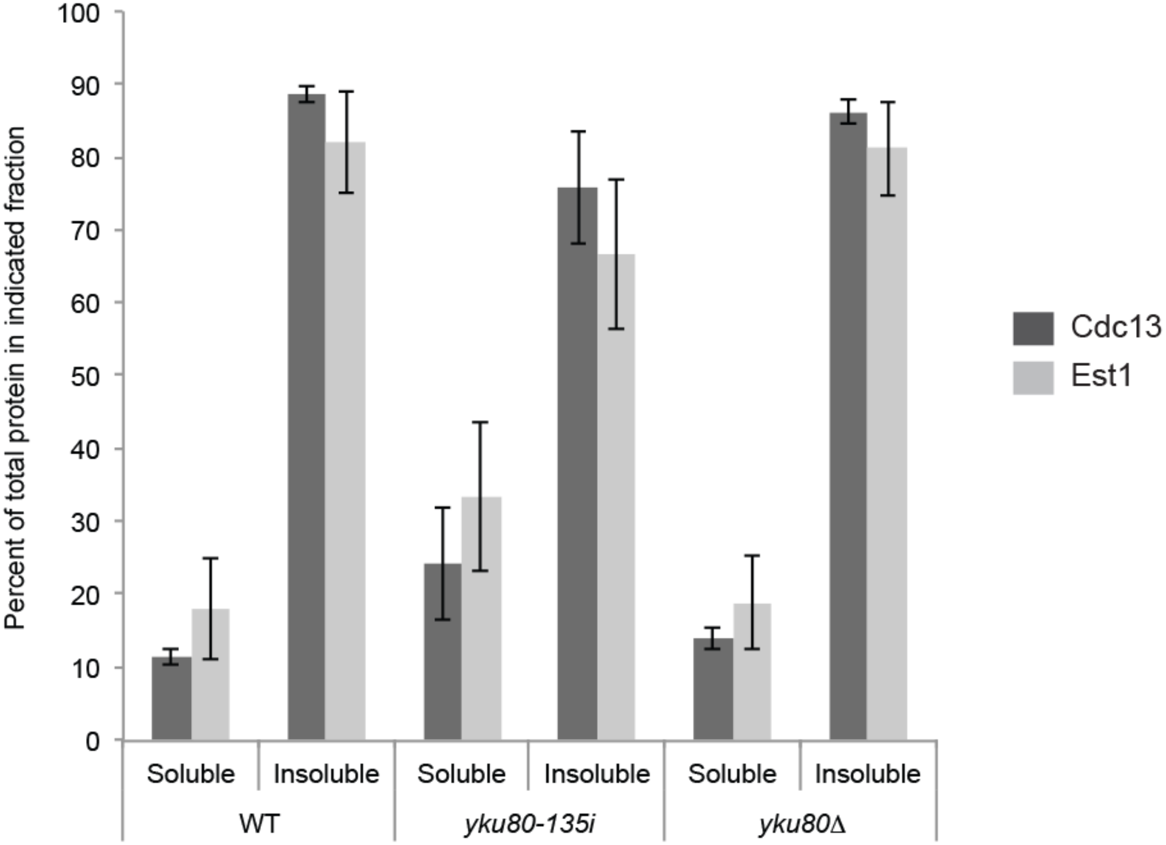

Supplementary Figure S5. The increased Est1:Cdc13 interaction is not caused by changes in protein solubility. Quantification of western blots for total Est1 and Cdc13 protein in soluble and insoluble fractions in three independent experiments. Indicated fractions were analyzed by western blotting with α-myc (Est1) and α-FLAG (Cdc13). Error bars represent ± 1 SEM.

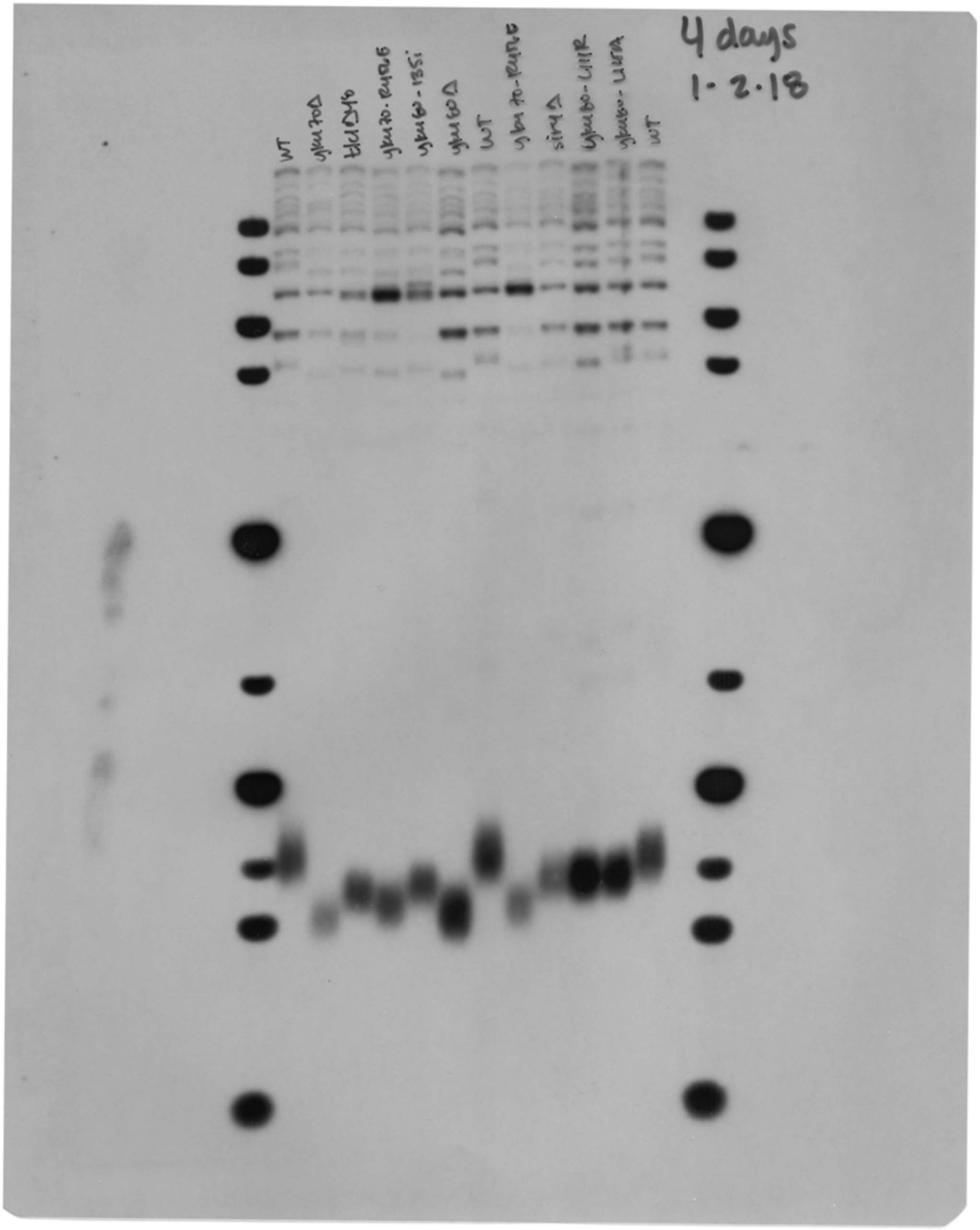

Supplementary Figure S6. Uncropped Southern blot shown in **Figure 1.**

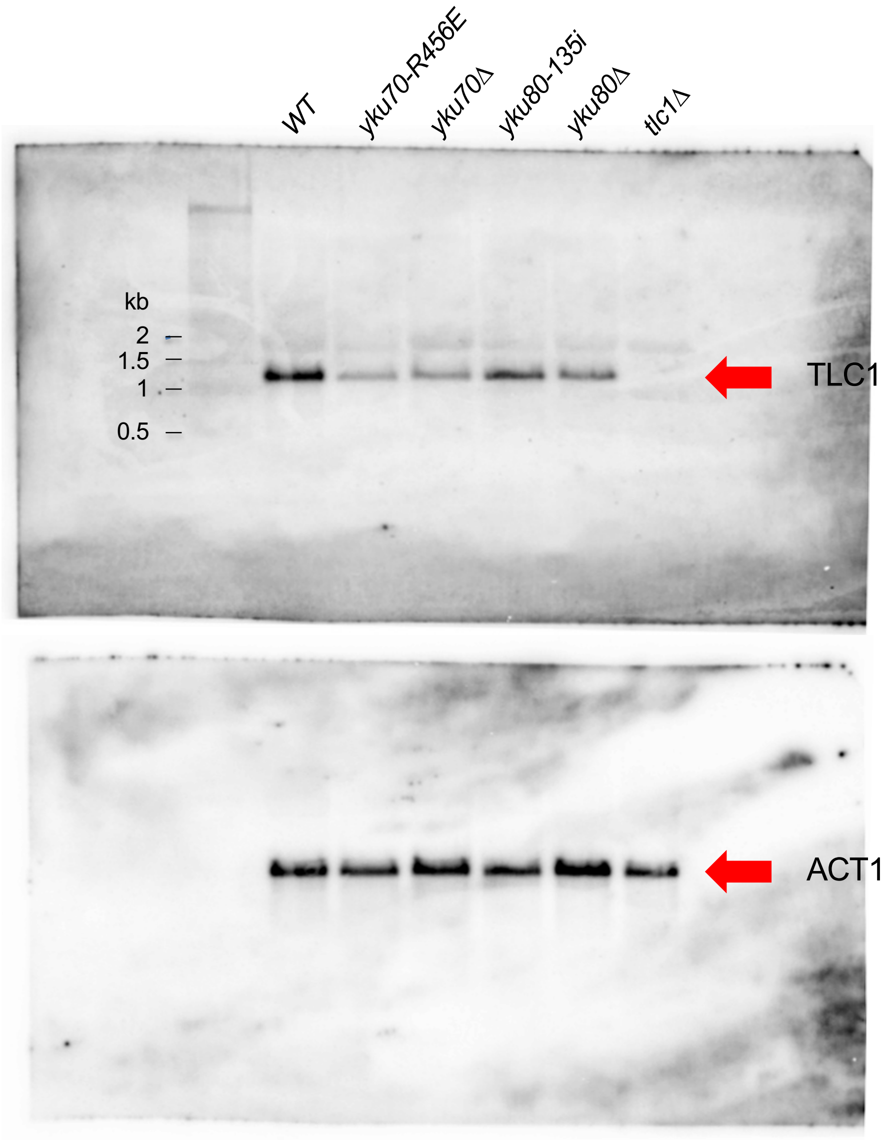

Supplementary Figure S7. Uncropped northern blots shown in **Figure 2B.**

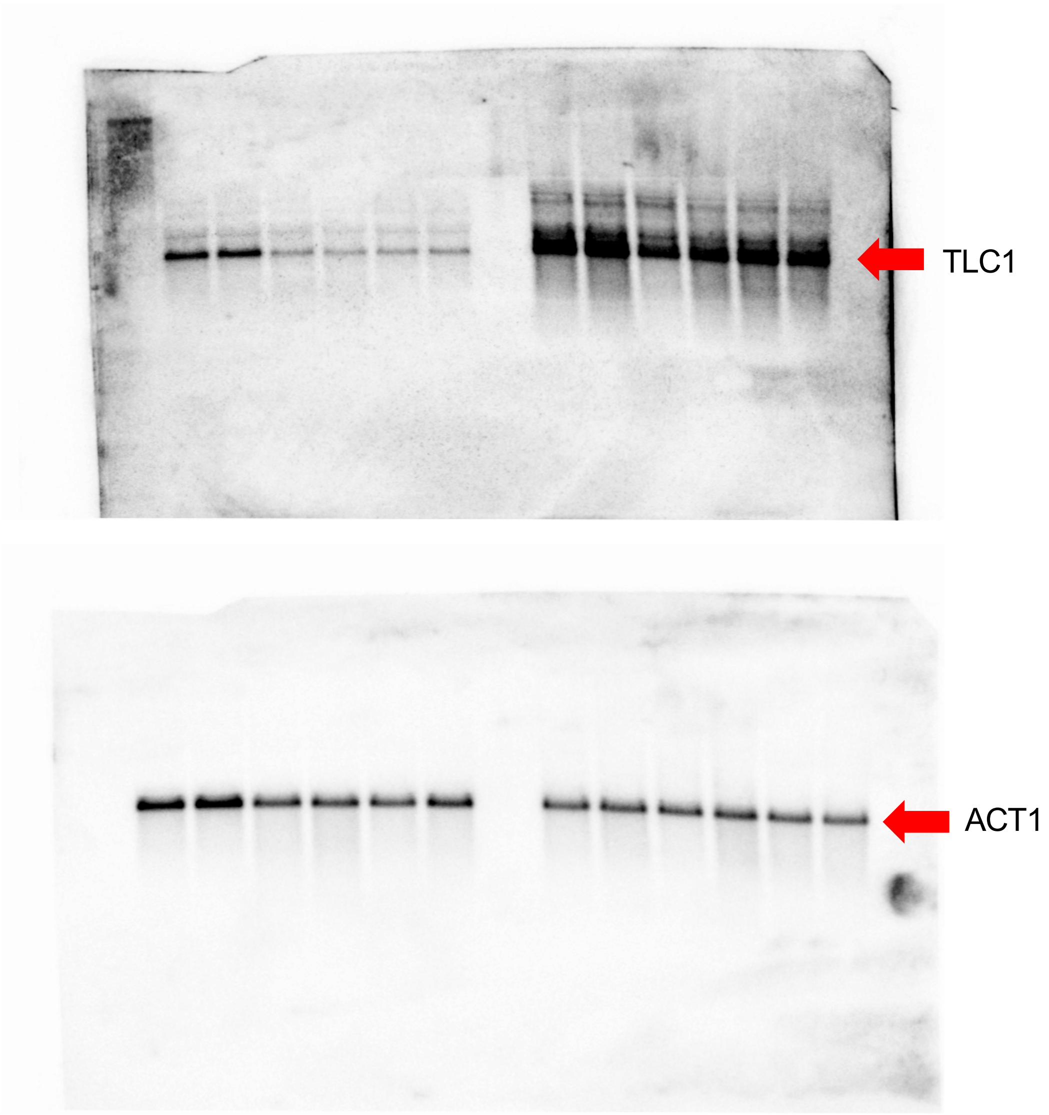

Supplementary Figure S8. Uncropped northern blots shown in **Figure 3A.**

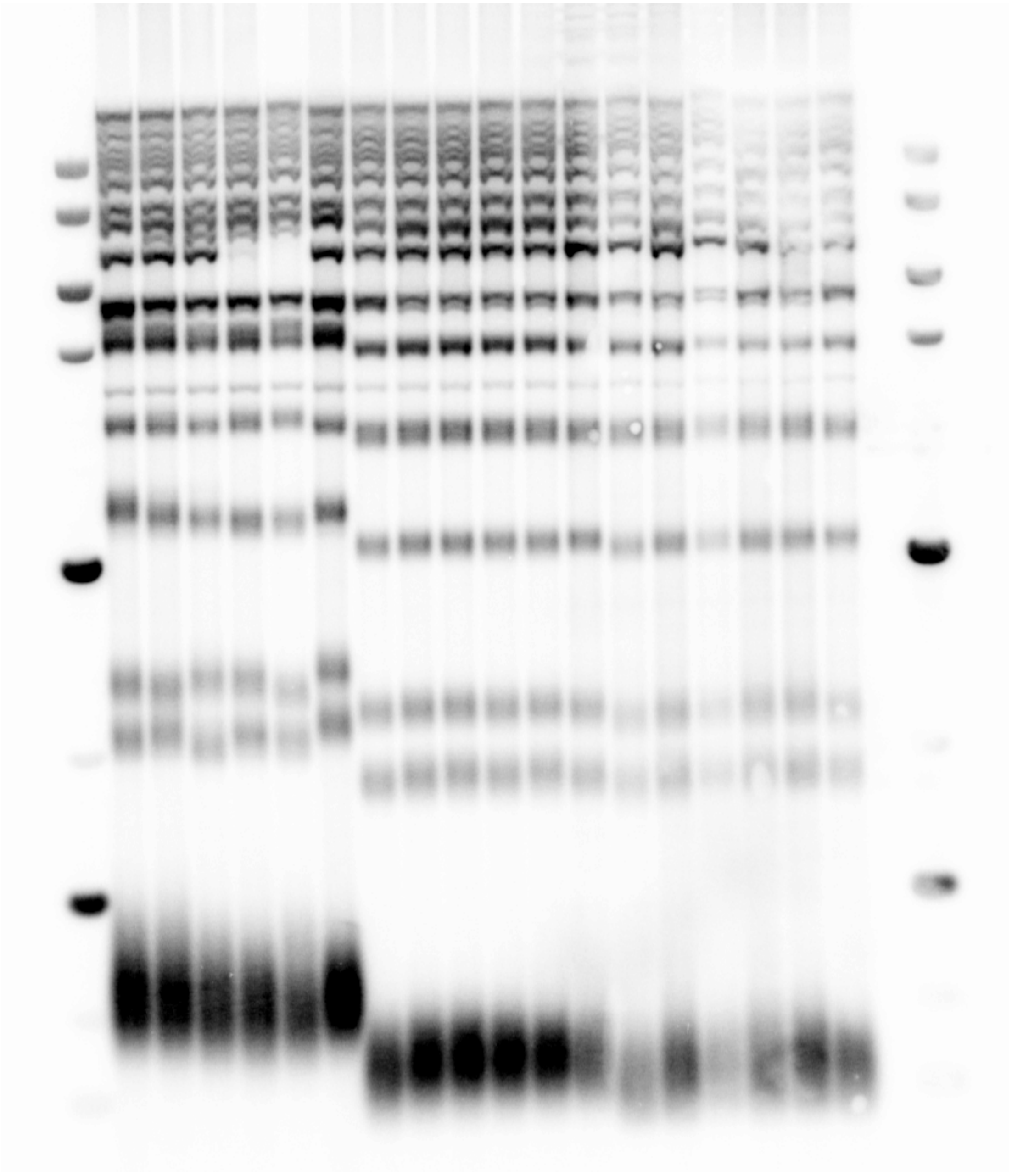

Supplementary Figure S9. Uncropped Southern blot shown in **Figure 3C.**

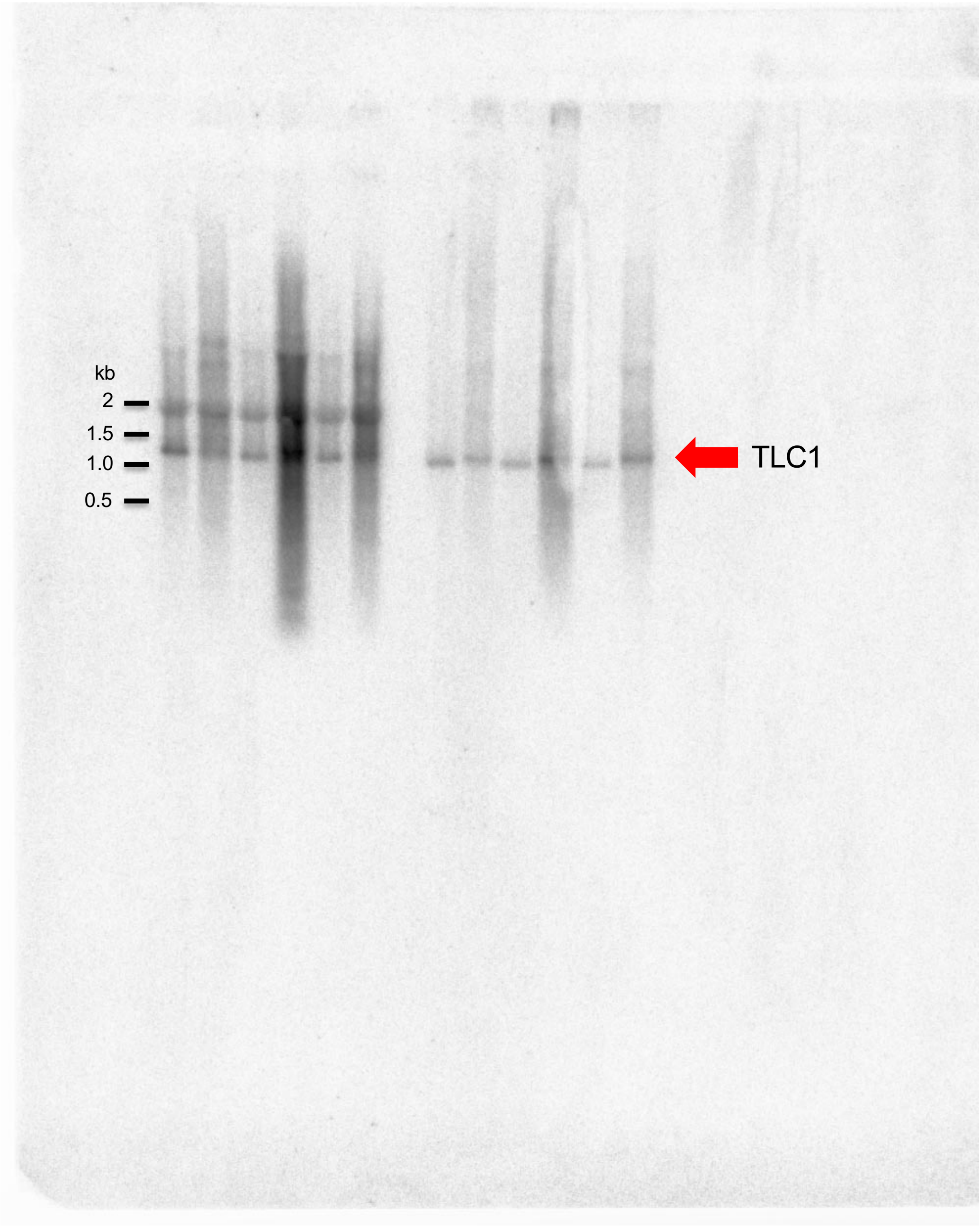

Supplementary Figure S10A. Uncropped TLC1 northern blot shown in **Figure 3D.**

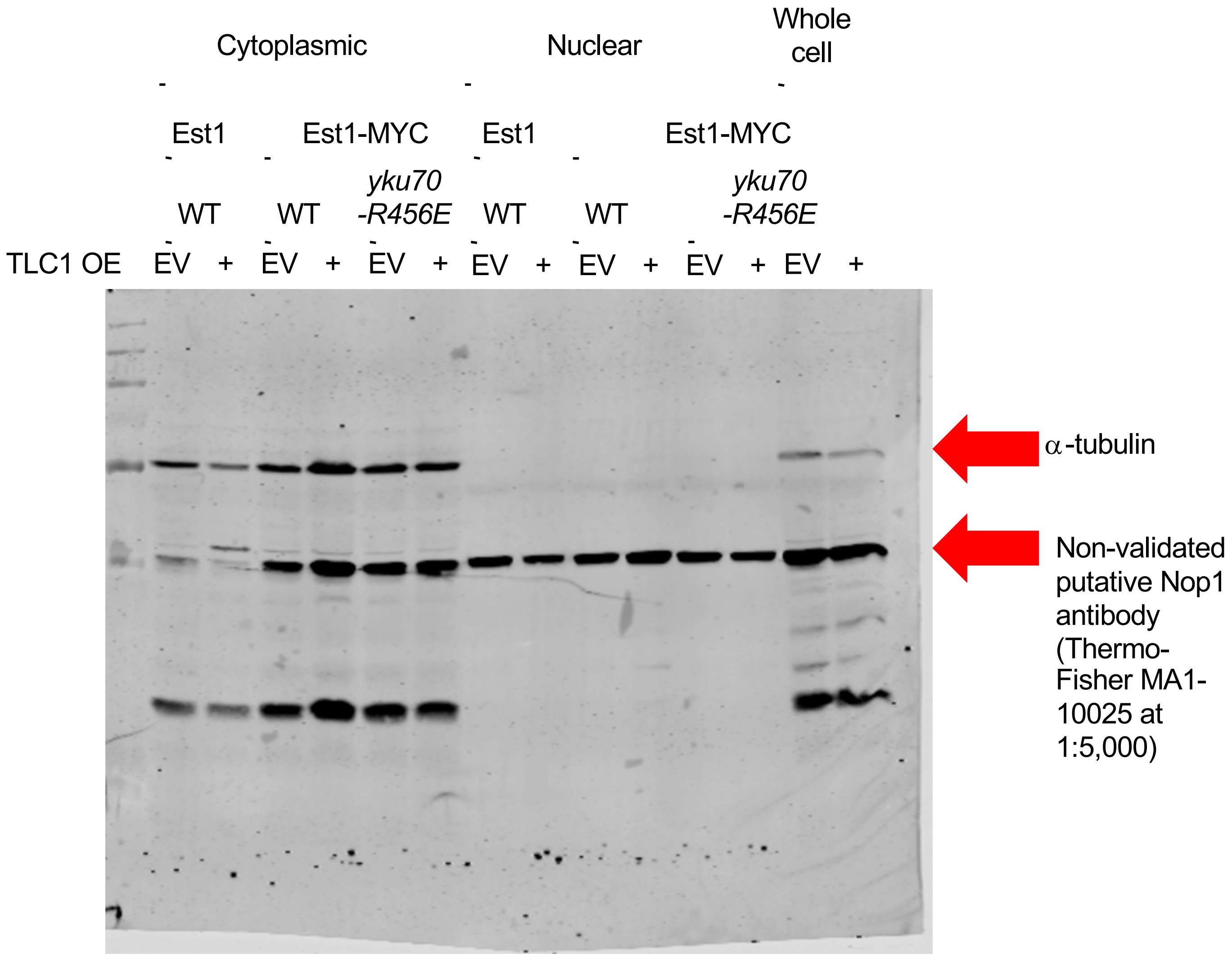

Supplementary Figure S10B. Uncropped western blot shown in **Figure 3D.**

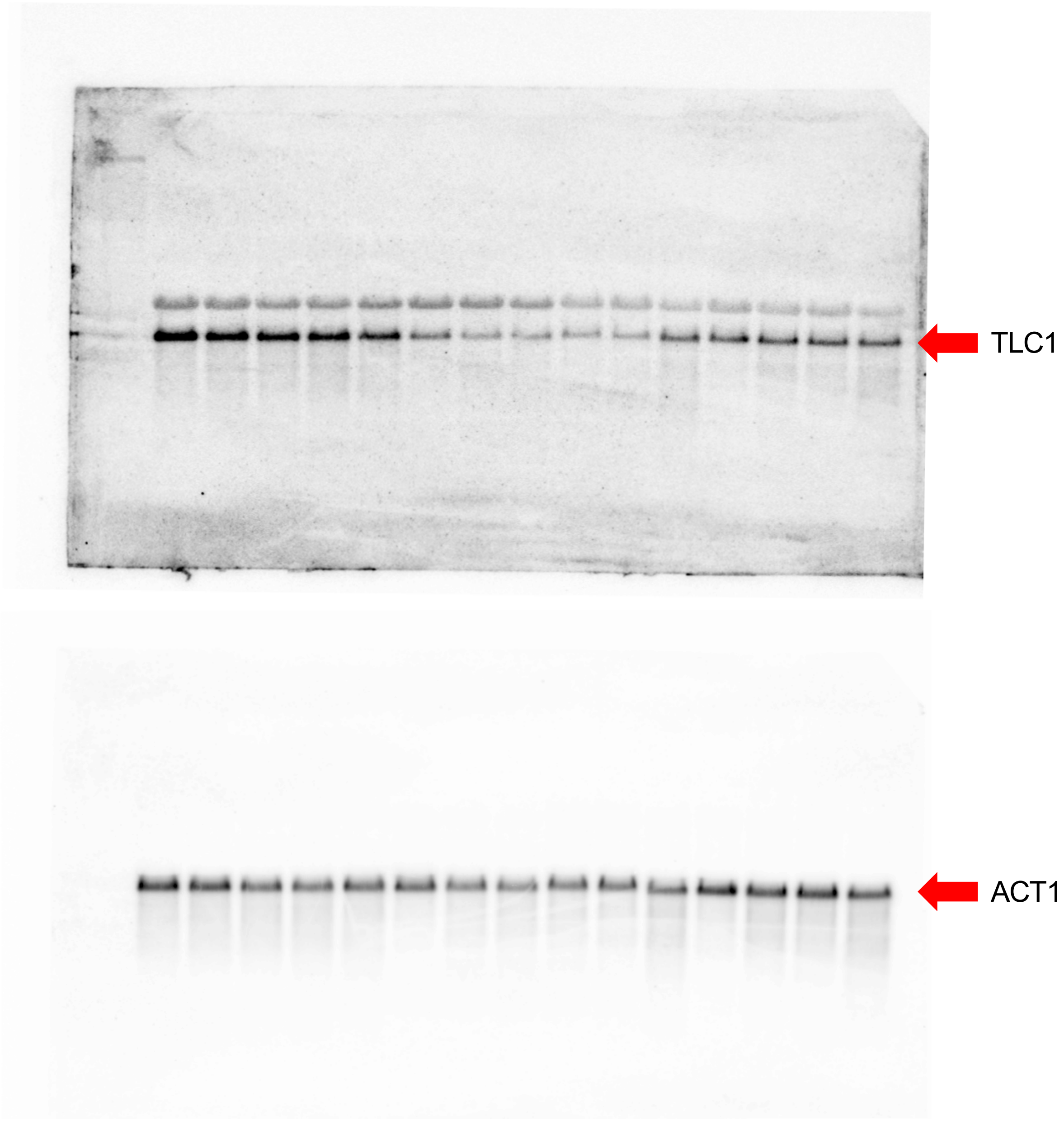

Supplementary Figure S11. Uncropped northern blots shown in **Figure 4A.**

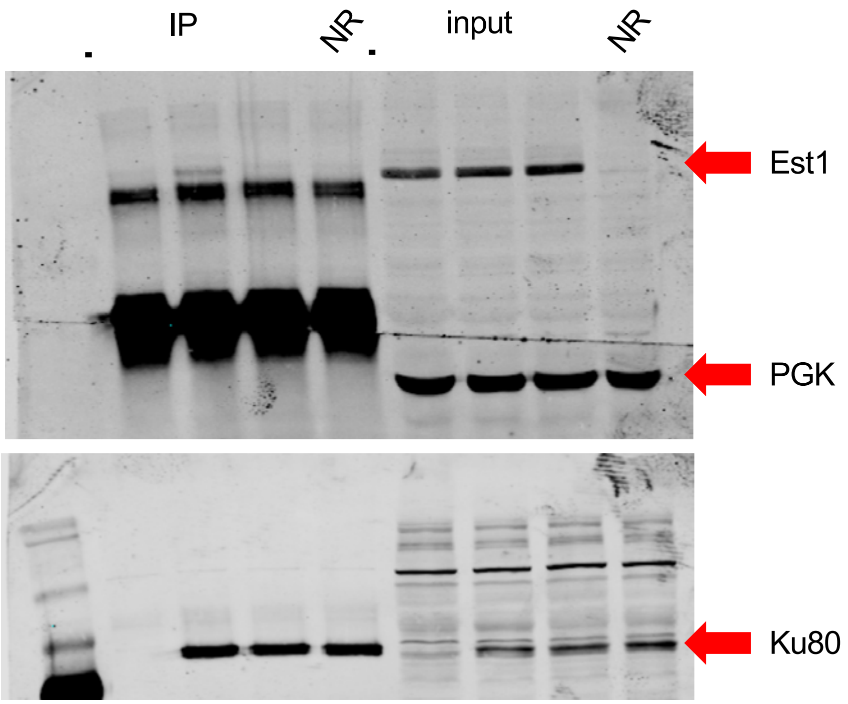

Supplementary Figure S12. Uncropped western blots shown in **Figure 5A**; NR indicates not relevant

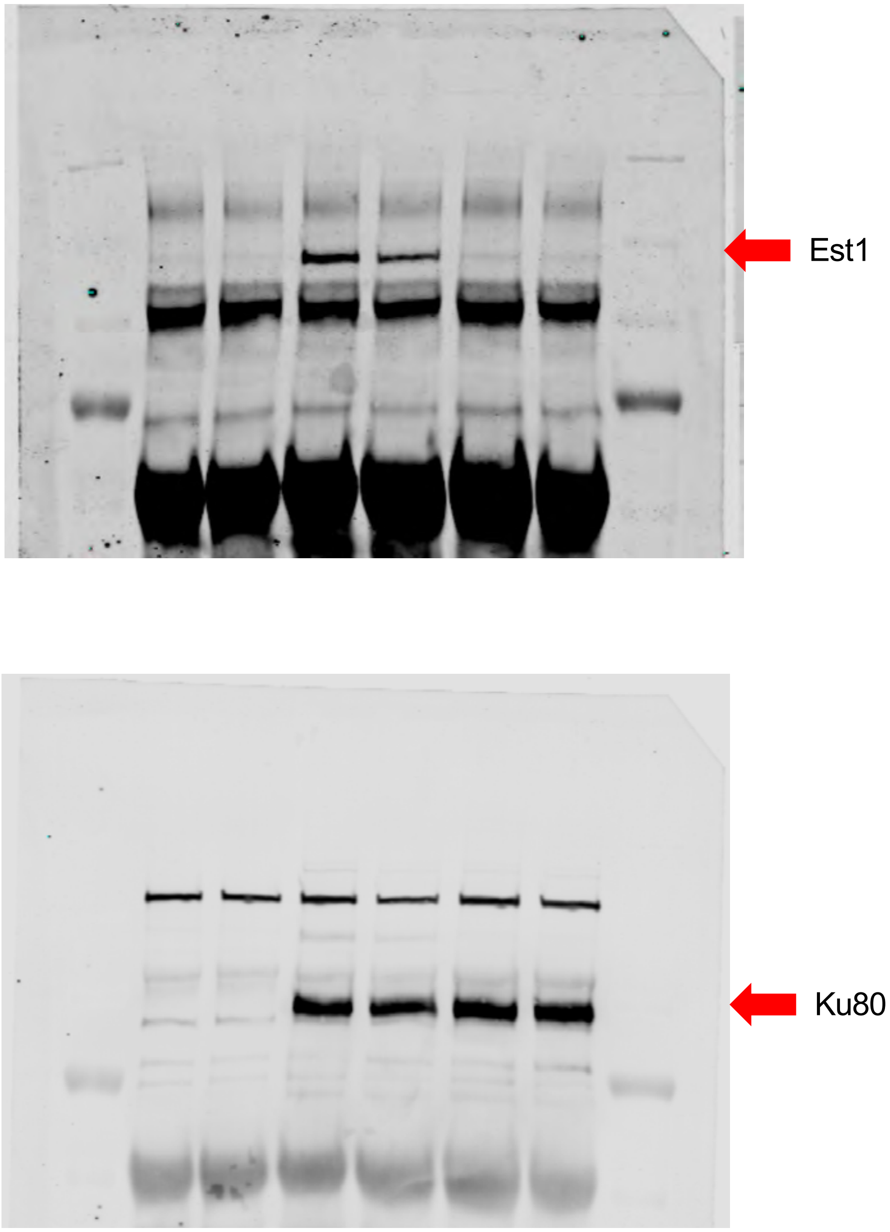

Supplementary Figure S13. Uncropped western blots shown in **Figure 5C.**

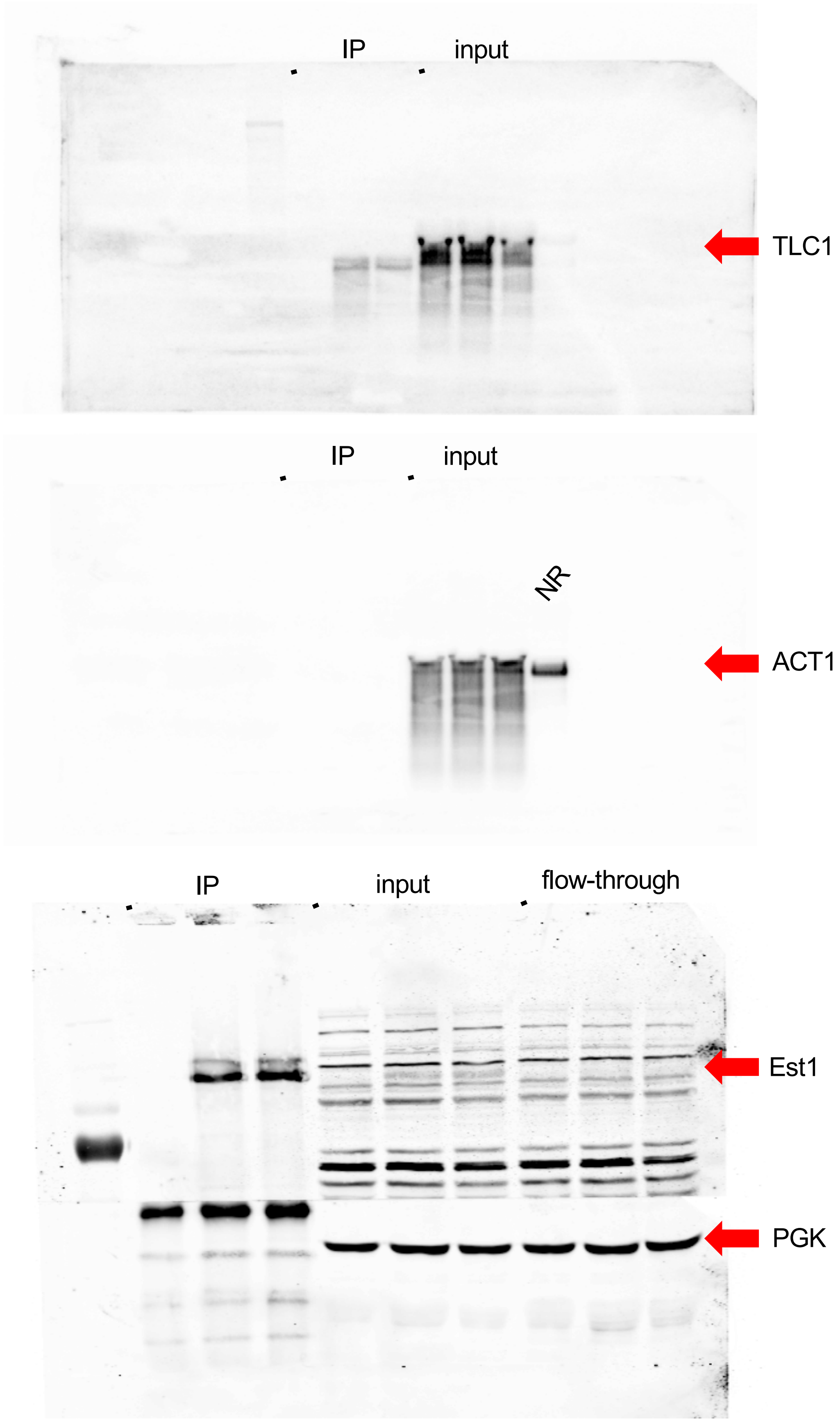

Supplementary Figure S14. Top: Uncropped northern blots shown in **Figure 5E**; NR indicates not relevant
Bottom: Uncropped western blots shown in **Figure 5E**

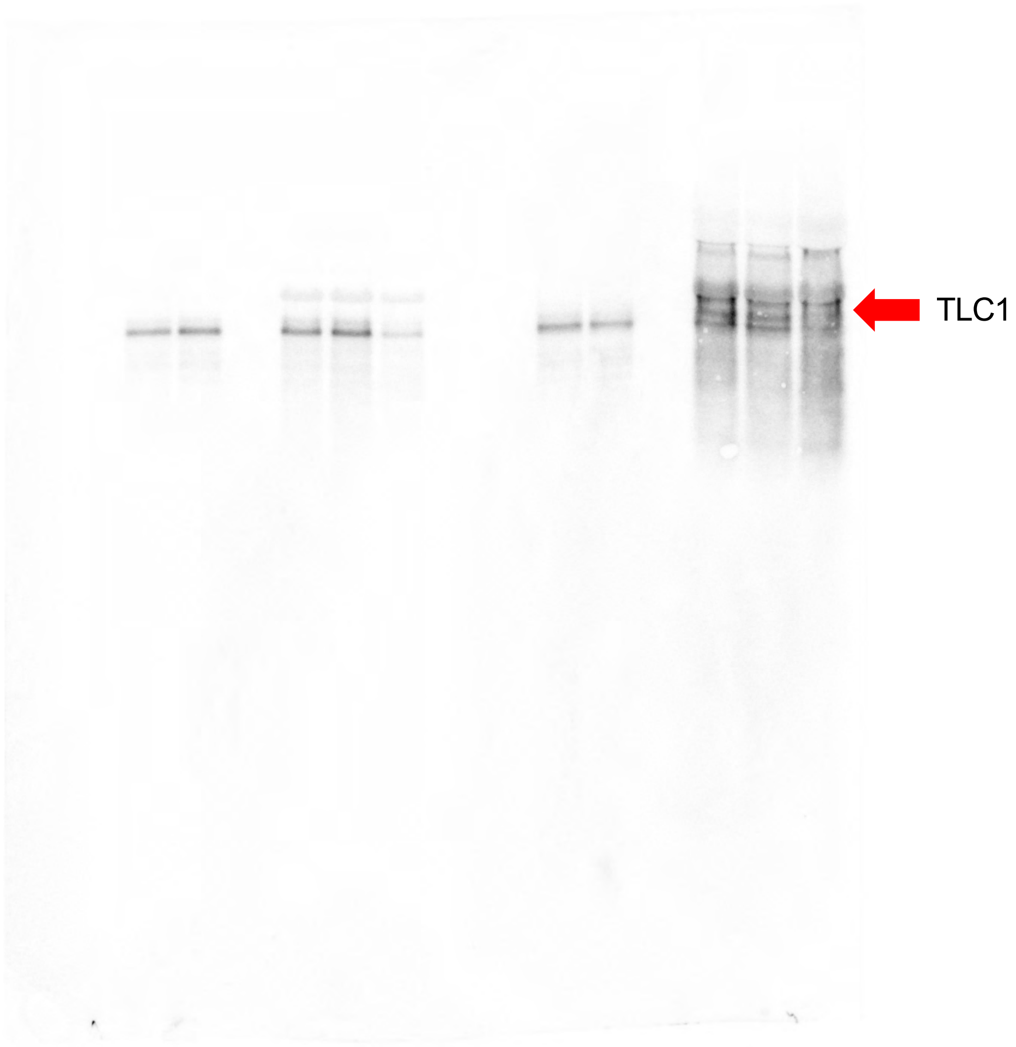

Supplementary Figure S15A. Uncropped northern blot shown in **Supplementary Figure S3,** which is representative of data quantified for **Figure 5H.**

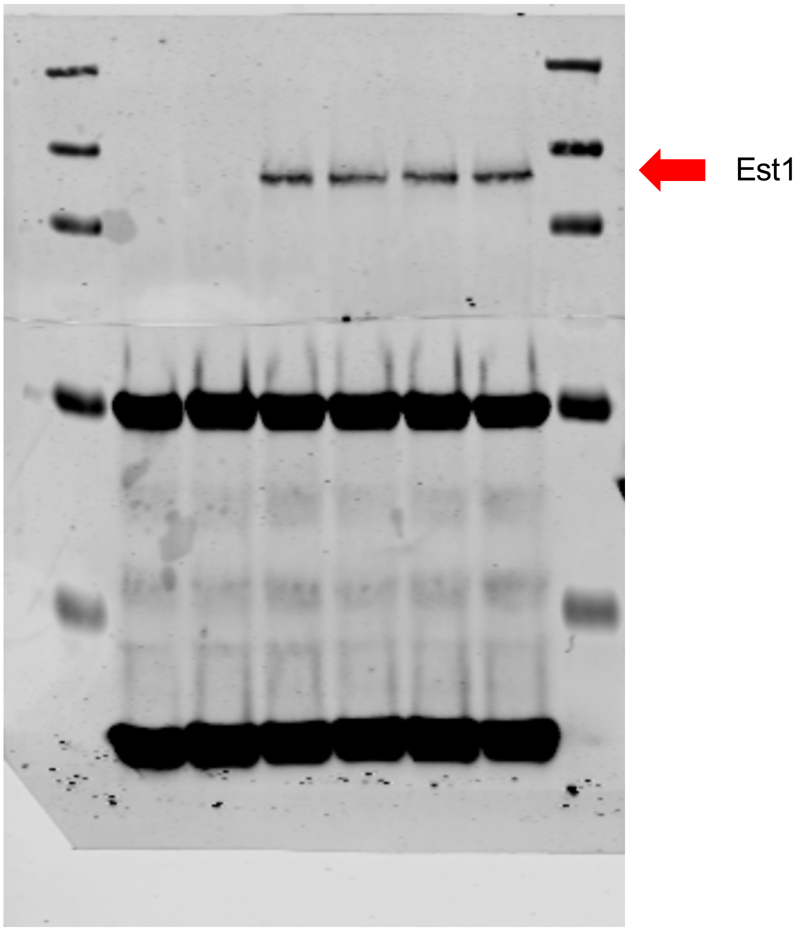

Supplementary Figure S15B. Uncropped western blot shown in **Supplementary Figure S3,** which is representative of data quantified for **Figure 5H.**

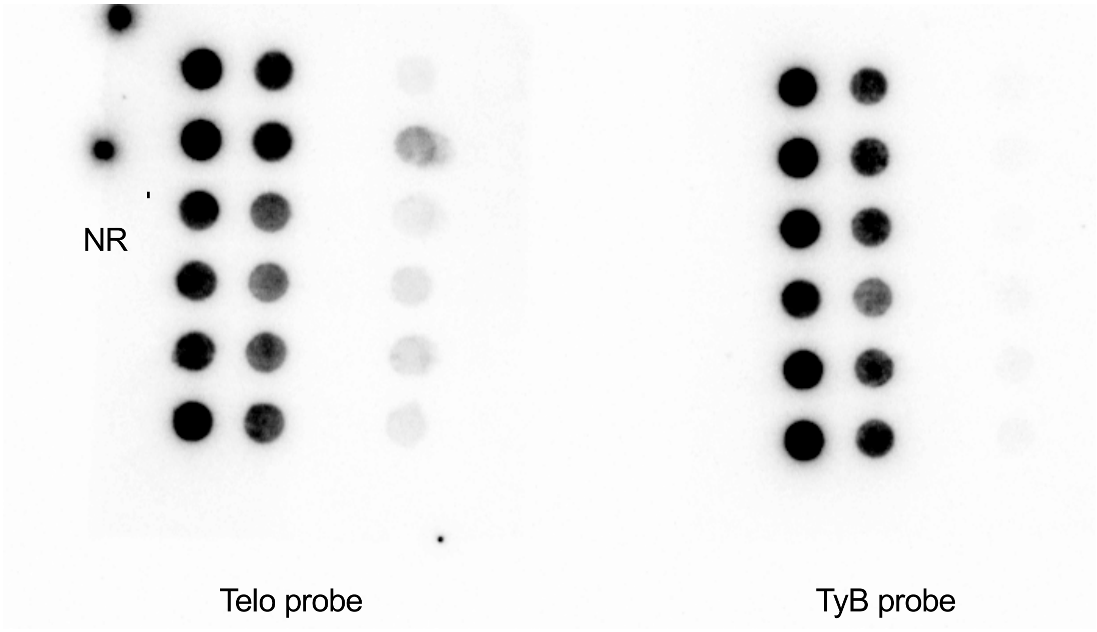

Supplementary Figure S16. Uncropped dot blots shown in **Figure 6A**; NR indicates not relevant.

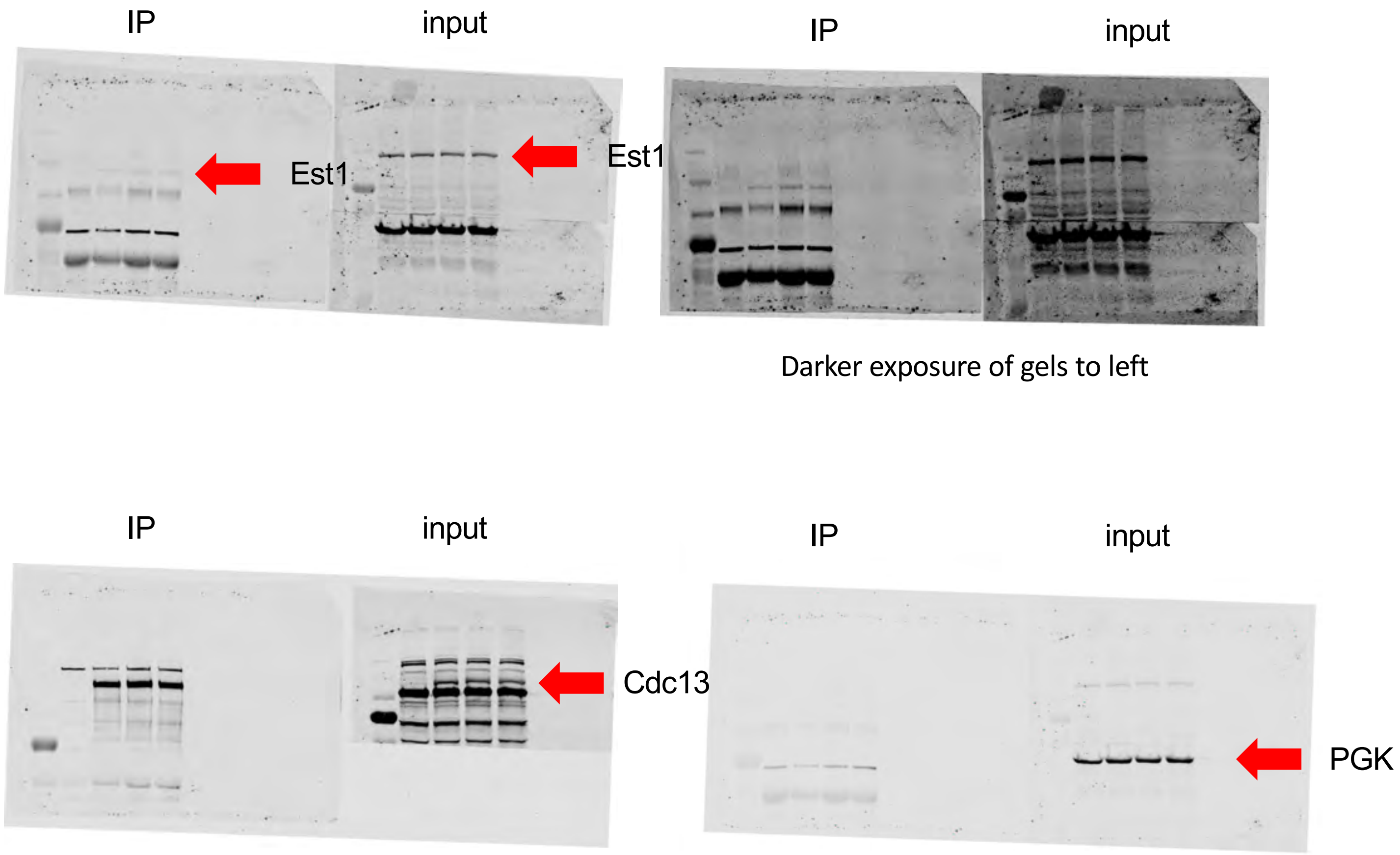

Supplementary Figure S17A. Uncropped top set of western blots shown in **Figure 7A**; NR indicates not relevant

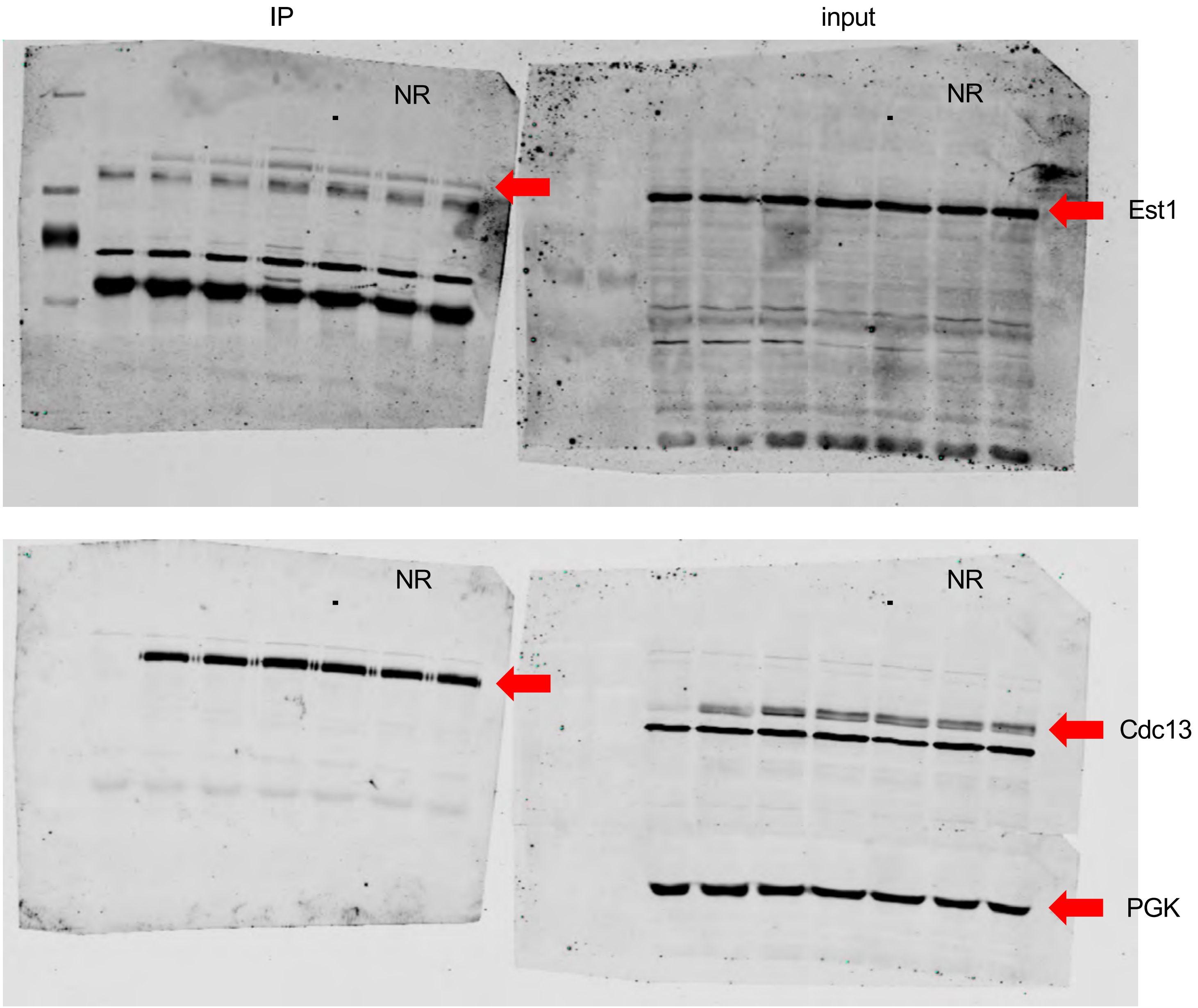

Supplementary Figure S17B. Uncropped bottom set of western blots shown in **Figure 7A**; NR indicates not relevant

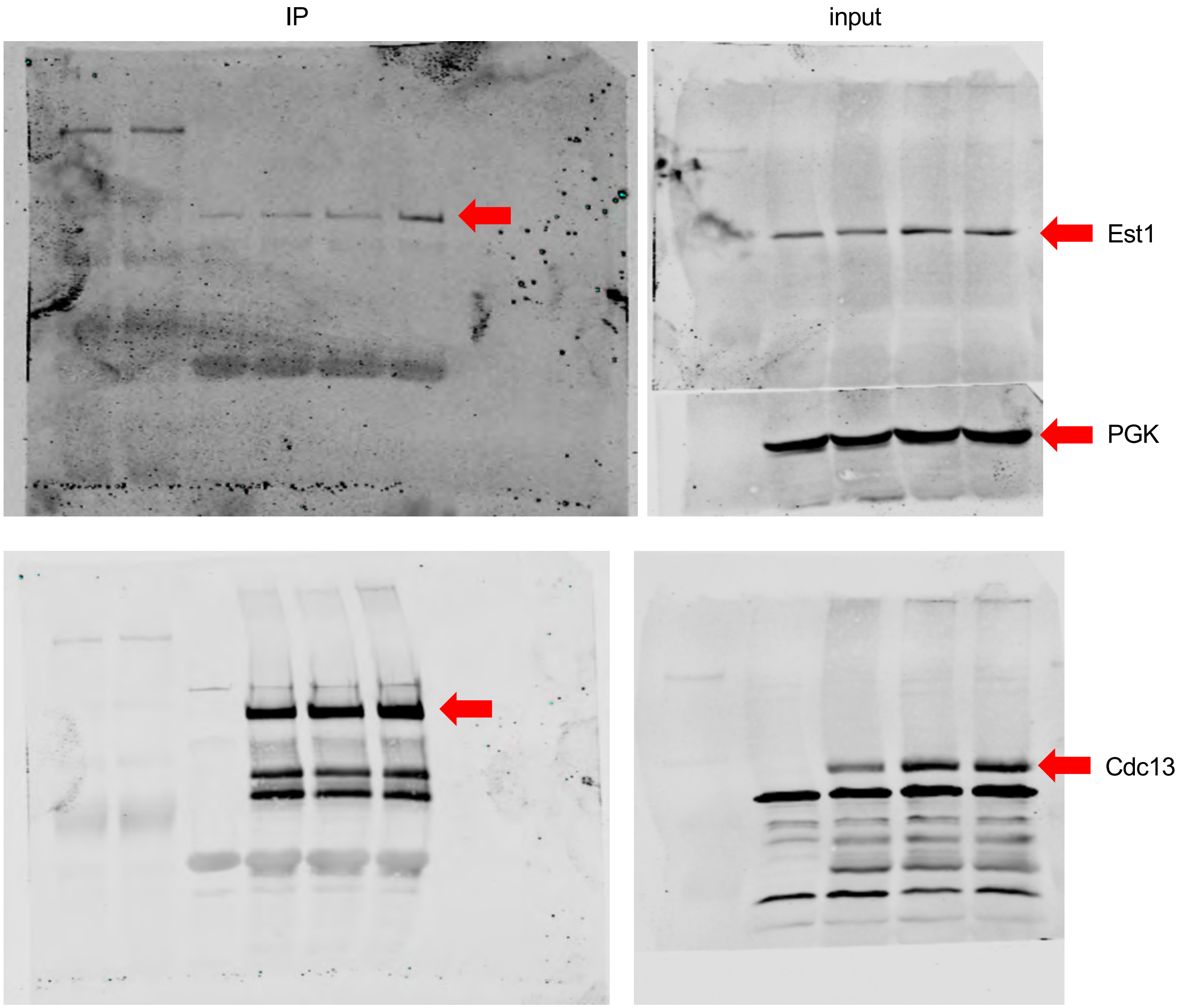

Supplementary Figure S18. Uncropped western blots shown in **Figure S4.**
